# Wide‐surface pore microfiltration membrane drastically improves sieving decay in TFF‐based perfusion cell culture and streamline chromatography integration for continuous bioprocessing

**DOI:** 10.1002/bit.27504

**Published:** 2020-07-30

**Authors:** Nuno D. S. Pinto, Mark Brower

**Affiliations:** ^1^ Process Research and Development, Merck & Co., Inc. Kenilworth New Jersey

**Keywords:** DNA sieving coefficient, integrated continuous bioprocess, membrane fouling, monoclonal antibody, tangential flow filtration perfusion

## Abstract

Although several compelling benefits for bioprocess intensification have been reported, the need for a streamlined integration of perfusion cultures with capture chromatography still remains unmet. Here, a robust solution is established by conducting tangential flow filtration‐based perfusion with a wide‐surface pore microfiltration membrane. The resulting integrated continuous bioprocess demonstrated negligible retention of antibody, DNA, and host cell proteins in the bioreactor with average sieving coefficients of 98 ± 1%, 124 ± 28%, and 109 ± 27%, respectively. Further discussion regarding the potential membrane fouling mechanisms is also provided by comparing two membranes with different surface pore structures and the same hollow fiber length, total membrane area, and chemistry. A cake‐growth profile is reported for the narrower surface pore, 0.65‐µm nominal retention perfusion membrane with final antibody sieving coefficients ≤70%. Whereas the sieving coefficient remained ≥85% during 40 culture days for the wide‐surface pore, 0.2‐µm nominal retention rating membrane. The wide‐surface pore structure, confirmed by scanning electron microscopy imaging, minimizes the formation of biomass deposits on the membrane surface and drastically improves product sieving. This study not only offers a robust alternative for integrated continuous bioprocess by eliminating additional filtration steps while overcoming sieving decay, but also provides insight into membranes' fouling mechanism.

## Graphical abstract

Although several compelling benefits for bioprocess intensification have been reported, the need for a streamlined integration of perfusion cultures with capture chromatography still remains unmet. The authors offer a solution, and further insight into membrane fouling, by using a wide‐surface pore microfiltration membrane as a TFF‐based cell retention device. The resulting integrated continuous bioprocess demonstrated negligible retention of antibody, DNA, and host cell proteins in the bioreactor with average sieving coefficients of 98%, 124%, and 109%, respectively.

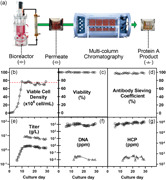

## 1 INTRODUCTION

Emerging capacity shortage and increased demand for cost‐appropriate therapeutic proteins is prompting a shift in biomanufacturing. Its conventional rigid stainless‐steel structure is being replaced with state‐of‐the‐art approaches, leveraging single‐use technologies as well as continuous and connected bioprocesses (Gagnon, Nagre, Wang, & Hiller, 2018; Klutz et al., 2015; Zydney, 2016). These more modern processes offer higher productivities, reduced facility footprint, and increased operational flexibility compared to the gold standard fed‐batch processes (Fisher et al., 2019 *)*. A cost‐attractive strategy for bioprocess intensification can be achieved by directly integrating high cell density perfusion cell cultures with capture chromatography (Pollock, Ho, & Farid, 2013; Somasundaram et al., 2018; Warikoo et al., 2012). However, this strategy puts the onus on the cell retention device to continuously harvest the bioreactor without product retention and provide a particle‐free permeate stream (Castilho & Medronho, 2002; Voisard, Meuwly, Ruffieux, Baer & Kadouri, 2003).

Biomanufacturing commonly employs hollow fiber membranes for cell retention in perfusion cell culture. Membrane fouling is the major challenge in these filtration‐based systems with known undesired product retention (product sieving) and ultimately membrane plugging. These challenges may lead to shortened manufacturing campaigns and/or reduced product recovery (Belfort, Davis, & Zydney, 1994; van Reis & Zydney, 2007). Besides perfusion operations, an additional challenge is the variable product concentration feeding the capture chromatography. The latter adds undesired complexity for bioprocess integration; it requires different loading volumes to maintain the target mass loading, and in turn to ensure a high yield and consistent product purity.

In the last decades, numerous literature studies focused on membrane fouling and product sieving in hollow fiber membranes (Mariorella, Dorin, Carion, & Harano, 1991; van Reis, Leonard, Hsu, & Builder, 1991; Stressman & Moresoli, 2008). Maintaining cell cultures with high viability is the major theme across several studies; this strategy avoids cell lysis and minimizes the release of potential membrane foulants. A few solutions listed, of particular interest for perfusion, were the addition of shear protectants in the cell culture media (Xu et al., 2017), the conservative perfusion crossflow range used (Karst, Serra, Villiger, Soos, & Morbidelli, 2016), and the deployment of low shear stress pumps (Blaschczok et al., 2013; Wang et al., 2017). Other studies focused on the comparison of perfusion technologies (Clincke, Mölleryd, Samani et al., 2013; Clincke, Mölleryd, Zhang et al., 2013), the unidirectional tangential flow filtration (TFF) and the alternating tangential flow (ATF). Although the ATF technology showed operational instability at higher cell densities and it did not solve the product sieving decay completely, it showed lower sieving decay than the TFF technology—suggesting that the bidirectional action minimizes biomaterial deposit onto the hollow fiber membrane. In addition, several mechanistic models of membrane fouling for both ATF and TFF technologies were developed (Bolton & Apostolidis, 2017; Kelly et al., 2014). These mathematical models suggest that biological material deposits onto the microfiltration membrane forming a cake that causes product sieving. Lastly, a parallel study by Wang et al. (2017) suggests that the biological material causing product sieving is comprised of particles within 20–200 nm range.

Our previous study (Pinto, Napoli, & Brower, 2020) showed an integrated continuous bioprocess with practically nonproduct retention when employing macroporous (>1 µm pore size) perfusion membranes. This observation, corroborated by Wang et al. (2017) and Wang et al. (2019), highlights that microfiltration membranes retain particles within its pore size range, which leads to product sieving. Whereas, submicron particles perfuse through the larger pores of macroporous hollow fiber membranes and thus overcome product sieving. Nonetheless, to remove particles from the perfusion permeate stream, an additional depth filtration step is required before the capture chromatography when employing macroporous membranes (Pinto et al., 2020). Therefore, a complete solution to overcome product sieving in hollow fiber membranes capable of yielding a particle‐free permeate has not been reported.

In addition to confirming that particles sized within the microporous membrane's nominal retention rating play a critical role in product sieving, our previous study also showed similar sieving behavior for both antibody and CHO genomic DNA molecules, namely, both family of molecules experienced retention in the bioreactor only with microfiltration membranes (Pinto et al., 2020). CHO genomic DNA present in cell culture broth has a wide particle size distribution including the range between ≈20 and 200 nm (Charlton, Relton, & Slater, 1998; Khanal et al., 2019). One hypothesis is that nucleic acids and/or CHO genomic DNA may be one of the constituents of the particles impacting product sieving. Mercille, Johnson, Lemieux, and Massie (1994) first explored this hypotheses by adding DNase I to the cell culture medium. Although the results were very encouraging by showing a drastic improvement in product sieving, this procedure is cost prohibitive at biomanufacturing scale.

The typical rejection rate is >90% for globular molecules with size larger than the membrane's nominal retention rating. Whereas, elongated biomolecules (such as DNA) demonstrate a high dependence on the orientation of the molecule. Tangential orientation with respect to the membrane leads to high retention. Normal orientation can be promoted by favorable permeate flux aligning the molecule's narrow side with the pore (Latulippe, Ager, & Zydney, 2007). This way, molecules can pass through the membrane pores even with several fold greater lengths than the membrane's nominal retention rating. Although a typical perfusion cell culture operates at permeate fluxes that are theoretically favorable of inducing DNA transmission (Latulippe et al., 2007), severe DNA sieving decay was reported (Pinto et al., 2020). In a potential pertinent study for perfusion cell culture, Li, Borujeni, and Zydney (2015) showed that DNA transmission is enhanced in asymmetric hollow fibers. These membranes induce pre‐elongation of DNA molecules through the membrane pores when entering the larger pore openings. This phenomenon enables elongation of molecules at lower fluxes. The study by Li et al. (2015) indicates a potential solution for product sieving and highlights an unexplored avenue in perfusion cell culture, that is, the role of the membrane's structure.

In this study, microfiltration hollow fiber membranes for perfusion cell culture were evaluated with the aim of assessing the impact of membrane's structure on product retention. Although several operating conditions were interrogated (i.e., crossflow shear rate and permeate flux), the membrane used was the most significant factor to overcome product sieving decay. Not only was antibody sieving decay drastically reduced, but a similar behavior was also detected for CHO genomic DNA when employing a wide‐surface pore membrane. Conversely, for the narrow surface pore membrane, and consistent with prior studies, the sieving decay was present for both antibody and DNA molecules. Lastly, for the first time, a successful bioprocess integration of the proposed solution to drastically improve product sieving decay and still yield a particle‐free permeate stream is shown. These results provide further insight into the membrane fouling mechanism in perfusion and demonstrate a feasible alternative to the commonly employed microfiltration membranes in integrated continuous bioprocesses.

## 2 MATERIALS AND METHODS

### 2.1 Perfusion cell culture

**Figure 1:**
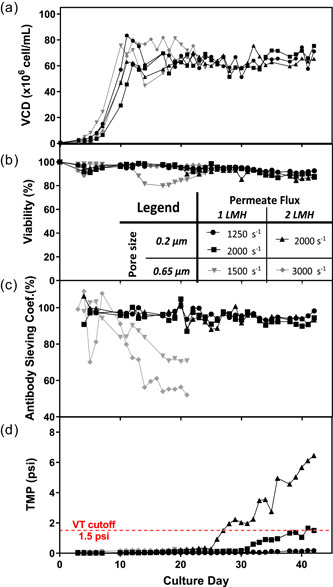
Tangential flow filtration‐based perfusion cell cultures with nominal retention ratings of 0.2‐µm in black (• 1,250 s^−1^ and 1 LMH, ▪ 2,000 s^−1^ and 1 LMH, ▴ 2000 s^−1^ and 2 LMH, crossflow shear rate and permeate flux, respectively) and 0.65‐µm in gray (▾ 1500 s^−1^ and 1 LMH, ♦ 3000 s^−1^ and 2 LMH, crossflow shear rate and permeate flux, respectively): (a) viable cell density (VCD); (b) viability; (c) antibody sieving coefficient; (d) transmembrane pressure (TMP). Increasing TMP indicates higher resistance for permeate flow, it was deemed high above the volumetric throughput (VT) cutoff of 1.5 psi (dash line) [Color figure can be viewed at wileyonlinelibrary.com]

In this study, the recombinantly produced monoclonal antibody was expressed in a CHO‐K1 cell line. Only glucose and antifoam were supplemented to the basal media, which is commercially available and chemically defined. Besides the 75 ± 10 × 10^6^ cells/ml target cell density maintained by discarding cell culture broth (bleed), all the other cell culture details were previously described by Pinto et al. (2020). Throughout this study, the perfusion technology employed relied on a Spectrum Laboratories KML‐100 TFF perfusion system (Repligen, Waltham, MA) with a centrifugal recirculation pump (Levitronix, Zurich, Switzerland) and a constant 0.75 vessel volume per day medium exchange rate. The membranes employed were the Asahi Kasei Microza 0.2 µm UMP‐1147R and 0.65 µm UJP‐1147R, both with 0.19 m^2^ surface area and made of polyvinylidene fluoride (Pall, Port Washington, NY). The perfusion operating conditions (crossflow shear rate and perfusion flux) for experiments performed in 3‐L glass bioreactors with 2 L working volume (Sartorius AG, Goettingen, Germany) are shown in the legend of Figure 1. With the notable exception for the 15‐L glass bioreactor, where a 8.5 L working volume (Sartorius AG) was used for the integrated continuous process demonstration.

### 2.2 Integrated continuous bioprocess demonstration

A TFF‐based perfusion cell culture was directly connected to the Cadence BioSMB (Pall). The perfusion was conducted with a Asahi Kasei Microza 0.2 µm UMP‐1147R membrane at a 1500 s^−1^ crossflow shear rate and 1.3 L·m^−2^·hr^−1^ permeate flux. The capture step utilized four OPUS prepacked chromatography columns with 1.2 cm i.d. × 10 cm length (Repligen, Waltham, MA) with MabSelect Sure resin (GE Healthcare, Marlborough, MA). The multicolumn capture chromatography step operated for 18 consecutive days, and it was initiated on 11th day of cell culture after a state of control had been established.

### 2.3 Analytical methods

Viable cell density (VCD) and viability were obtained by Cedex HiRes analyzer (Roche, Mannheim, Germany). Reverse‐phase high‐performance liquid chromatography was employed for titer measurements. Both residuals, DNA via quantitative polymerase chain reaction and host cell protein (HCP) by enzyme‐linked immunosorbent assay, were determined using methods developed at Merck & Co., Inc. (Kenilworth, NJ).

Unused 0.2 and 0.65 µm membranes were flushed with water for injection and drained by gravity. Sections of approximately 4 inches in length extracted from the filter modules were then frozen in liquid nitrogen and cut with a sharp razor. A Hitachi S‐3400 scanning electron microscope (SEM), with a fixed 5‐kV acceleration voltage, was used to characterize the topographies of the 0.2 and 0.65 µm membranes at ×5,500 and ×20,000 magnifications, respectively.

### 2.4 Hydraulic permeability

The experimental setup for hydraulic permeability measurements with purified water was the same as the perfusion cell culture experiments. The hydraulic permeability (*L_p_*) was determined by the slope of the following equation (Zeman & Zydney, 1996):

(1) $$J_{v}=L_{p}\times \text{TMP}.$$

The measurements consisted in varying the crossflow flowrate, enabling adjustments to the transmembrane pressure (TMP), and the corresponding permeate flux (*J_v_*) was determined by timed collection.

In addition, the computational equations described in our previous study (Pinto et al., 2020) were also employed here to determine the sieving coefficient, TMP, wall shear rate in the TFF membrane, as well as the perfusion volumetric productivity.

## 3 RESULTS

### 3.1 Perfusion membrane assessment

The initial focus of this study is to evaluate two membranes for cell retention in TFF‐based perfusion cell culture. To allow a clear interpretation of the role of each membrane on product sieving, both membranes were procured from the same manufacturer with matching hollow fiber length, chemistry, and filtration area. The two remaining key membrane characteristics taken in consideration for the membrane selection were the nominal retention rating and membrane structure. First, the nominal retention ratings purposely chosen within the microporous range (0.2 and 0.65 µm) ensure a particle‐free permeate stream—note that in absence of membrane's structure information, the larger 0.65‐µm retention rating may seem the most likely option to minimize sieving decay. Second, different surface pore membrane structures allow to probe the hypothesis that the transmission of submicron particles, leading to product sieving, can be enhanced by the membrane structure. This way, providing insight toward the mechanism of membrane fouling and, at the same time, offer a solution to drastically reduce product sieving decay.

Five cell cultures interrogating both 0.2 and 0.65 µm perfusion membranes, as well as several operating conditions, are shown in Figure 1. The operating ranges studied include crossflow shear rates between 1,250 and 3,000 s^−1^ and permeate flux rates between 1 and 2 L·m^−2^·hr^−1^, which are commonly employed in perfusion cell culture. Here, highly viable cultures targeting 75 × 10^6^ cells/ml of VCD were successfully maintained for 42 and 21 days with 0.2 and 0.65‐µm perfusion membranes, respectively (Figure 1a,b). The two 0.65‐µm perfusion cultures were terminated when the antibody sieving coefficient was ≤70% and with a minimum of 3 weeks of operation (Figure 1c).

All perfusion cultures conducted with the 0.2‐µm Microza membrane show antibody sieving coefficient >85% throughout 40 days (Figure 1c), independent of the crossflow shear rates and permeate fluxes studied. In contrast, lower antibody sieving coefficients are shown in Figure 1c for the 0.65‐µm perfusion cultures. The latter is speculated to follow a cake‐growth profile as reported by Bolton and Apostolidis (2017). This profile suggests that biomaterial deposits onto the perfusion membrane until a balance of forces between the tangential sweeping action and normal drag toward the membrane surface results in the formation of a stable gel layer. The final constant sieving coefficient, indicative of a stable gel layer, was higher at the lower crossflow shear rate, namely, 71 ± 1% and 55 ± 2% corresponding to a shear rate of 1,500 and 3,000 s^−1^, respectively. Although the higher crossflow flowrate increases the tangential sweeping action, here it led to a lower and less desirable final antibody sieving coefficient. A conceivable membrane fouling mechanism, supporting the data presented, is that a higher crossflow velocity reduces polarization of the antibody, reducing the transmission by lowering the antibody concentration at the upper surface of the cake layer. These observations, constant final sieving, and worse sieving decay with increased shear rate, are in line with results from other commonly employed microfiltration membranes for TFF‐based perfusion (see Appendix A1).

**Figure 2:**
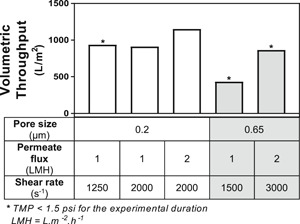
VT comparison relative to membrane's pore size (i.e., supplier's nominal retention rating), permeate flux, and crossflow shear rate based on the 1.5‐psi transmembrane pressure cutoff shown in Figure 1d. VD, volumetric throughput

In addition to sieving coefficient decay, membrane fouling may lead to full pore blockage reducing the available surface area for filtration. In the case of pore blockage, in order to maintain the constant permeate flow, the TMP will increase to overcome the higher resistance, which ultimately leads to an operational failure. A maximum 1.5 psi TMP criteria (dash line in Figure 1d) was used to determine the operational volumetric throughputs that can be tolerated in production, shown in Figure 2. The small difference between the 0.2 µm Microza membrane (900–1,200 L/m^2^) volumetric throughputs is not only consistent with previous microfiltration perfusion reports (Clincke, 2017), but also demonstrates a robust performance within the range of conditions studied. Here, both 2,000 s^−1^ crossflow shear rate conditions demonstrated an TMP increase ≥1.5 psi, whereas the 1,250 s^−1^ condition did not show a TMP increase. Nonetheless, the full impact of crossflow shear rate on volumetric throughput is not clear, since the experiment was terminated on Day 42 of cell culture before all conditions had reached the criteria. Additionally, the volumetric throughputs for the 0.65‐µm membrane were within 450–900 L/m^2^. The perfusion experiments conducted with 0.65‐µm membranes would also have achieved higher loadings according to the criteria; however, loadings were determined on Day 21 when the batches were terminated due to high antibody sieving decay (≤70%).

**Figure 3:**
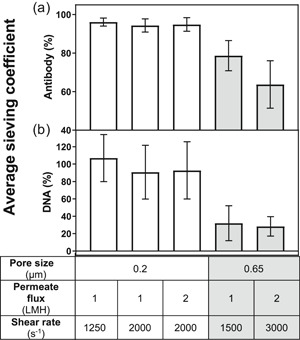
(a) Antibody and (b) DNA average sieving coefficients for the perfusion cell cultures shown in Figure 1 (error bars correspond to one standard deviation)

**Figure 4:**
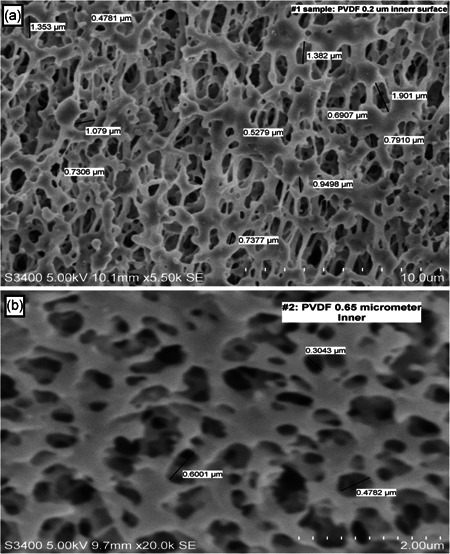
Scanning electron microscopy topographies of the Microza membranes: (a) 0.2‐µm UMP and (b) 0.65‐µm UJP with ×5,500 and ×20,000 magnifications, respectively

Antibody and DNA average sieving coefficients were both reduced only when employing the 0.65‐µm membrane (Figure 3). This finding confirms our previous (Pinto et al., 2020) observation that DNA and antibody transmissions are similarly impacted. Moreover, the 0.2‐µm Microza membrane showing a nearly complete transmission for antibody and DNA is a remarkable result for perfusion cultures conducted with microfiltration membranes. To provide further insight into the mechanism of fouling, the inner lumen side of both membranes was imaged by SEM and the topographies are shown in Figure 4. The range of surface pore diameters observed were 0.5–2 µm and 0.3–0.6 µm for the 0.2 µm and 0.65 µm membranes' nominal retention rating, respectively. Although unexpected, hydrodynamic permeability (*L_p_*) measurements confirm the lower nominal retention rating of the 0.2‐µm membrane even though a wider surface pore structure is observed.

With the membrane hydraulic permeability (shown in Table 1) and assuming uniform cylindrical pores, the mean pore radius (*r_p_*) was determined using the equation as follows (Zeman & Zydney, 1996):

(2) $$r_{p}={\left(\frac{8\mu \delta_{m}L_{p}}{\varepsilon }\right)}^{1/2},$$

where μ is viscosity, $\delta_{m}$ is membrane thickness, and ε is the membrane porosity. The pore diameter shown in Table 1 was determined using Equation (2) and the experimentally measured *L_p_*, assuming ε = 0.5, μ = 1 cP = 1.45 × 10^−7^ psi·s (water viscosity), and $\delta_{m}$ = 0.04 cm (full membrane thickness). Good agreement between the observed surface pore diameter, pore diameter determined from *L_p_* data, and the nominal retention rating for the 0.65‐µm membrane suggests a membrane structure with cylindrical pores (Table 1). Contrastingly, the wide‐surface pore diameter observed for the 0.2‐µm Microza membrane is in disagreement with both the pore diameter determined from *L_p_* data and its nominal retention rating. For instance, the permeability of a membrane with uniform cylindrical pores of 1.25 µm diameter (average surface pore diameter observed) would be ~fourfold greater than a 0.65‐µm membrane according to Equation (2), since the permeability is related to the square of the pore radius. However, Table 1 shows the opposite permeability result, where *L_p_* of 0.65‐µm membrane is ~fourfold higher than the 0.2‐µm Microza membrane. Thus, the wide‐surface pore and lower permeability suggest a complex membrane structure that may be described by a conical rather than cylindrical pore structure.

**Table 1:** Microfiltration membrane's hydraulic permeability (*L_p_*) and comparison between membrane's nominal retention rating, pore size diameter determined from permeability data, and the observed surface pore diameter range from SEM imaging (shown in Figure 4)

Microfiltration membrane nominal retention rating (µm)	Hydraulic permeability (*L_p_*)^a^ (cm·s^−1^·psi^−1^)	Pore size diameter using *L_p_* & Equation (2) (µm)	Observed surface pore diameter range via SEM (µm)
0.2	3.08 × 10^−3^	0.34	0.5–2.0
0.65	1.14 × 10^−2^	0.65	0.3–0.6

Abbreviation: SEM, scanning electron microscopy.

a Hydraulic permeability determined by fitting Equation (1) to experimental data with *R* ^2^ ≥ 0.98.

Purification by TFF of DNA molecules in simple matrixes—without the complexity of perfusion cell culture with cells, microvesicles, exosomes, wide range of soluble proteins, and nucleotides—may help elucidate the high DNA and antibody transmission obtained when using the 0.2‐µm Microza membrane. In TFF, DNA transmission is induced when operating above the critical permeate flux (*J*_crit_; Latulippe et al., 2007). The critical flux for symmetric membranes with cylindrical pores is calculated from Equation (3) (Daoudi & Brochard, 1978):

(3) $$J_{\text{crit}}≅\frac{\varepsilon \;k_{B}T}{{\mu r}_{p}^{2}},$$

where $k_{B}$ is the Boltzmann constant and *T* is temperature. The theoretical critical flux (*J*_crit_) for the 0.2‐ and 0.65‐µm membranes are 51 and 5 µm/s, respectively, assuming ε = 0.5, μ = 1 cP, and *T* = 295K. More recently, Latulippe et al. (2007) showed that Equation (3) overpredicts *J*_crit_ by three orders of magnitude, the improved *J*_crit_ model yielded a *J*_crit_ = 0.1 µm/s for a ~73‐nm plasmid DNA in a 0.22‐µm membrane. The permeate flux employed during the perfusion experiments were (~0.3 and ~0.6 µm/s corresponding to 1 and 2 LMH, respectively) above the *J*_crit_ predicted by Latulippe et al. (2007), indicating high theoretical DNA transmission for both membranes. Nonetheless, the notably high antibody and DNA transmission for the 0.2‐µm Microza membrane is a remarkable result in perfusion cell culture. The complex cell culture broth matrix may require lower *J*_crit_ to induce DNA transmission than predicted by Latulippe et al. (2007) due to the presence of a wide range of molecular sizes.

The unique membrane structure of the 0.2‐µm membrane, with wide‐surface pores and low permeability, may be approximated by conical shaped pores. The critical flux for a conical pore structure can be approximated by (Li et al., 2015):

(4) $$J_{\text{crit}}^{\prime }=(D/\delta_{m})J_{\text{crit}},$$

where *D* is the diameter of larger opening of the conical pore. Assuming *D* = 1.25 µm (Figure 4a), the average pore size (*D*) observed, and $\delta_{m}$ = 400 µm, Equation (4) yields *J*′_crit_ 320 folds lower than the *J*_crit_ for a cylindrical pore. Operating significantly above *J*′_crit_ may induce pre‐elongation of DNA molecules through the membrane pores when entering the larger pore openings and may significantly improve the DNA transmission. These results not only demonstrate that the membrane structure plays a critical role in product sieving, but also that overcoming DNA retention may be an appropriate strategy to drastically improve product sieving decay—as suggested by Mercille et al. (1994).

### 3.2 Integrated continuous bioprocess with a wide‐surface pore microfiltration perfusion membrane

To assess the robustness of integrated continuous bioprocesses when employing wide‐surface pore membranes (e.g., 0.2‐µm TFF Microza membrane, see Section 3.1), a perfusion cell culture was directly connected to a continuous capture step without any additional filtration. A potential purification challenge that could arise from this approach is column fouling. Not only the potential of column collapsing due to backpressure generated from particles in the permeate stream accumulating at the top of the column, but also additional dissolved impurities present as a result of the improved sieving coefficient, namely DNA and HCPs.

**Figure 5:**
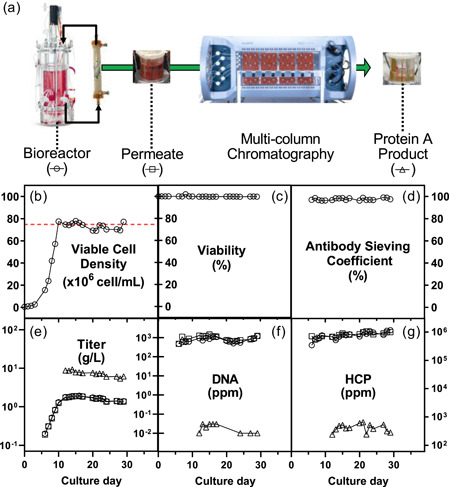
Integration of a continuous capture chromatography (Protein A) with a perfusion cell culture conducted with a 0.2‐µm Microza membrane: (a) schematic illustration of the process integration and sampling locations (dotted line); (b) VCD; (c) viability; (d) antibody sieving coefficient; (e) antibody concentration (titer); (f) residual CHO genomic DNA; and (g) host cell proteins. Note that the dash line in panel (b) corresponds to the setpoint for cell density control and the bottom panels y‐axis (e–g) are in logarithmic scale with base 10. VCD, viable cell density [Color figure can be viewed at wileyonlinelibrary.com]

The schematic illustration of the integrated continuous process is shown in Figure 5a. The TFF‐based perfusion was controlled with an average VCD, viability, and volumetric productivity of 74 ± 3 × 10^6^ cells/ml, 99.7 ± 0.1%, and 1.2 ± 0.2 g·L^−1^·day^−1^, respectively, after Day 10 of cell culture (Figure 5b,c). The narrow standard deviation on the VCD control yielded a highly viable cell culture with consistent productivity. In addition, the TFF membrane not only operated with TMP ≤ 0.5 psi throughout the 29 culture days, but retention of soluble components was negligible with average sieving coefficients of 98 ± 1%, 124 ± 28%, and 109 ± 27% for antibody, DNA, and HCP, respectively. These observations are in full agreement with the 3‐L perfusion cultures (Section 3.1). Lastly, the capture purification step operated consistently for 18 days without signs of column fouling, namely stable feed pressure, as well as constant DNA and HCP reductions (Figure 5e–g). The logs of reduction for DNA and HCP of >4 and >3, respectively, are in line with industry's practice.

## 4 CONCLUSIONS

This study shows that wide‐surface pore perfusion membranes are capable of overcoming product sieving decay in perfusion cell culture. For the first time in TFF‐based perfusion, a microfiltration membrane is not impacted by submicron‐sized particles that commonly cause membrane fouling. In traditionally employed perfusion membranes, particles within ~20–200 nm may deposit onto the membrane surface since these are too small to be swept away by the crossflow tangential forces. In contrast, the wide‐surface pore perfusion membrane's structure induces the transmission of polymeric biomolecules present in perfusion cell culture broths (e.g., DNA molecules). Hence, a particle‐free permeate stream is obtained with a drastically improved product sieving decay. This way, the perfusion connection to the capture step is streamlined and demonstrated in a 29‐day long integrated continuous bioprocess. These results not only offer a robust alternative for integrated continuous bioprocess that eliminates the need for additional filtration steps, but also provide further insight of the fouling mechanism in perfusion membranes.

## Figures and Tables

**Figure A1:**
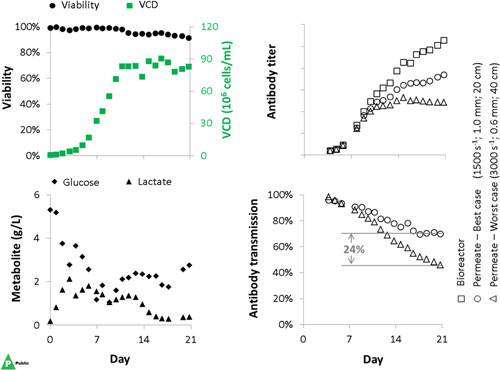
Perfusion employing two shear stress conditions. Best case: 1,500 s^−1^ wall shear rate, hollow fiber with 1‐mm diameter and 20‐cm length. Worst case: 3,000 s^−1^ wall shear rate, hollow fiber with 0.6‐mm diameter and 40‐cm length [Color figure can be viewed at wileyonlinelibrary.com]

## APPENDIX A

### Impact of shear rate in TFF‐based perfusion cell culture

Herein, a single 3‐L perfusion cell culture is conducted with two TFF perfusion systems to interrogate the shear rate impact on antibody sieving. This experimental setup intentionally removes the impact of cellular damage of two TFF systems (at high and low wall shear rates) by using the same bioreactor. This way, the profiles for the antibody sieving coefficient obtained assess solely the impact of filtration conditions on the membrane fouling. With the intent of finding the shear rate that balances the effects between minimal cell lysis and high cleaning action, the shear rates employed corresponded to the limits suggested by Mariorella et al. (1991), namely 1,500 and 3,000 s^−1^. In light of Vickroy, Lorenz, and Kelly (2007) work, where cell lysis in hollow fibers was found to be proportional to the magnitude of shear stress and residence time in the lumens, the additional membrane characteristics taken in consideration were the lumen's length and diameter. The worst case with longer and narrower lumens operated at the highest crossflow shear rate (3,000 s^−1^), whereas the best case was conducted with opposing conditions, with a S04‐M20U‐06‐N and S02‐M20U‐10‐N (Repligen, Waltham, MA), respectively.

Figure A1a shows an average steady state VCD of 83 × 10^6^ cells/ml and 94% cell culture viability over the last 11 days of cultivation. After 21 days of cell culture (Figure A1d), the minimum antibody transmission was 70% and 46% for the best and worst cases, respectively. Although, both membranes are connected to the same bioreactor and therefore being fed the same foulant concentration, a 24% antibody sieving coefficient difference is observed. Such an outcome, between low (1,500 s^−1^) and high (3,000 s^−1^) wall shear rates, suggests that the biomass deposited on the membrane surface is more compressed due to higher crossflows, which, in turn, increases the resistance for antibody transmission.
